# To assess the contributing factors of nutritional and health status amongst elderlies residing in the nursing homes of fars province, Iran: A cross sectional study

**DOI:** 10.1002/hsr2.1940

**Published:** 2024-03-07

**Authors:** Hassan Joulaei, Parisa Keshani, Seyed Mohammadsadegh Kashfinejad, Zohre Foroozanfar, Mohammad Ali Mohsenpour, Mohammad Fararouei

**Affiliations:** ^1^ Health Policy Research Center, Institute of Health Shiraz University of Medical Sciences Shiraz Iran; ^2^ Department of Epidemiology Shiraz University of Medical Sciences Shiraz Iran; ^3^ HIV/AIDS Research Center, Institute of Health Shiraz University of Medical Sciences Shiraz Iran; ^4^ Student Research Committee Shiraz University of Medical Sciences Shiraz Iran

**Keywords:** elderly, health status, malnutrition, Mini Nutritional Assessment, need assessment, nursing house

## Abstract

**Background and Aims:**

In line with the global trend, the number of elderly is rising in Iran. It should be noted that the nutritional and health needs of these people, especially those living in nursing houses, are extremely significant. The present study aimed to identify the nutritional and health status and uncover their relationship with received care services by elderlies residing in nursing homes.

**Methods:**

All 373 elderlies living in the nursing homes in Shiraz entered the study, and the requisite data were collected by references to elderlies' files, respective nurses, and elderlies themselves.

**Results:**

The majority of the elderlies were at risk of malnutrition (62.5% of the total population, 55.4% of females, and approximately 44.6% of males). Elderlies with no insurance coverage mainly fell into the at‐risk and malnutrition categories. There was a significant relationship between the needs assessment scores, reflecting the need for physical and psychological actions, and elderlies' malnutrition (odds ratio = 1.87, 95% confidence interval: 1.79−1.95). For each unit increase in the Physical and psychological need score, the odds of exposure to malnutrition (vs. nonmalnourished and at risk of malnutrition) was 1.87 times after adjustment for confounders.

**Conclusion:**

The results of our study revealed that most of the elderlies living in the nursing homes of Shiraz had some levels of malnutrition such as at‐risk for malnutrition (62.5%) and malnourished (18.9%). Based on these results, it is suggested that health policy‐makers take practical steps toward promoting the nutritional care of old people and direct extra supervision to nursing homes and all healthcare provisions.

## 1 INTRODUCTION

Aging is one of the fundamental and critical periods in the lifespan perspective. This period is accompanied by declines in physical, biological, and sometimes mental strengths.^1^ Although the population aged 60 and over has been defined as “Elderly,” it can be varied from one country to another based on age, socioculture, and functional status.^2^ For instance, the elderly term is defined in developed countries as aged 65 years or above, while in some countries retirement age (aged 50, 60, or 65 years), or special physical condition is considered for its definition.^3^ With the development and expansion of health sciences, the number of elderly increases annually, and the world moves toward agedness rapidly. Adults over 60 made up 11% of the population in 2010, and it is predicted to dramatically rise up to 21.2% in 2050.^4^ Iran also shows a similar trend, in which the elderly population increased from 5% to 8% from 1980 to 2015, and is expected to show a fourfold growth and reach 31% of the population by 2050.^5^

Usually, aging is accompanied by decreased quality of life and increased diseases causing infirmity, especially physical activity‐related illnesses. It can be lead to a loss of independence in daily activities and reduce the ability to manage personal affairs which are the main concerns in old age. It is believed that quality of life is highly dependent on the quality of services provided by health and social service institutions, such as nursing homes.^6^

Elderlies residing in nursing homes are at risk of malnutrition,^7^ for both micro‐^8^ and macro‐nutrient intake.^9^ It should be noted that malnutrition, which can reduce the elderly's operational condition is associated with poor health conditions and high mortality rates. Also, it is one of the chief problems of the elderly living in nursing homes.^6^ Malnutrition‐suffering elderlies are exposed to several health‐related risks, including falling, long hospitalization periods, postsurgery side effects, infection, gastric ulcers, long wound‐healing periods, and muscular and respiratory disorders.^10^

Physical activity also affects the quality of life among elderlies.^11^ Moreover, physical activity can positively contribute to supplying the nutritional health of old people.^12^ Oral health, as another quality life‐related factor in the elderly, is also affected during aging.^13^ Of these conditions, dental problems and saliva reduction cause poor oral health and increase the risk of tooth decay and infections.^13^, ^14^ A study on the oral health conditions of elderlies in Arizona revealed that the average number of existing teeth was 18.15, the average of decayed filled teeth was 28.86, and the average root decay index equaled 15.23. This study also showed that receiving care services at home or being hospitalized in a nursing center considerably predicted poorer conditions of oral health concerning the crown and root cavities.^14^

Individuals' mental health is another condition that is deleteriously affected by aging. In a study conducted in European countries, Andreas and his coworkers showed that one out of two old‐aged people have suffered from mental disorders during their lives. They also showed, that one out of three individuals was got involved in mental disorders during the year before the study. Affective disorders and substance abuse‐related conditions along with anxiety were the most prevalent disorders among them.^15^

In line with the global trend, the number of elderly is rising in Iran. Thus, considering the importance of the nutritional and health needs of these vulnerable people, especially those living in nursing homes, the present study aimed to identify their nutritional and health status and uncover their relationship with received care services to enlighten reliable evidence for relevant policy‐makers and their health status.

## 2 MATERIAL AND METHODS

### 2.1 Study design and population

The present cross‐sectional study was conducted first to assess the nutritional and health status of elderly dwelling nursing homes, and then find their possible relationships with the received care services. Out of 9 nursing homes in Fars province, Iran, seven were located in Shiraz, and 2 were in other cities in the Fars province. All residents in seven nursing homes of Shiraz were included in the study which led to the participation of 373 elderlies dwelling in these nursing homes.

### 2.2 Data collection

The requisite data of elderlies whom were resided in the nursing homes and agreed to participate in the study were collected by references to elderlies' files, respective nurses, and elderlies themselves. Also, by reviewing the medical records, the participants' demographic and medical data, including age, marital status, anthropometric indices, blood pressure, education level, self‐perceived health condition, oral health, appetite, illnesses, used medicines, symptoms, last blood tests, as well as the latest dates in which elderlies' have been visited by a general practitioner, specialist, nutritionist, psychologist, and psychiatrist were collected by nurses and extracted from their healthcare documents.

### 2.3 Malnutrition assessment

The malnutrition status of elderlies was assessed by the valid Mini Nutritional Assessment (MNA) questionnaire. The MNA investigates the malnutrition risk with seven subscales of appetite and eating discomfort, weight loss, physical ability, acute mental problem, psychological problem, body mass index (BMI), and calf circumference. Individuals are classified for the risk of malnutrition with the score of the MNA tool as normally nourished, a score of 12−14, at risk of malnutrition with a score of 8−11, and known as malnourished if scored 7 or less. This questionnaire was completed with the help of a direct nurse of the elderly.

### 2.4 Physical and psychological assessment

The Physical and Psychological Need (PPN) questionnaire is the shortened format of the Camberwell Assessment of Need Questionnaire which was used for examining the psychological status of participants. The Camberwell scale was shortened concerning the purpose of the study and validated by a panel of six experts. The final questionnaire included 13 items and was scored on a 4‐point Likert scale (strongly disagree, disagree, moderately agree, strongly agree). Items of this questionnaire asked about the elderly's need for personal care, memory, visual, hearing, physical activity, anxiety, stress, self‐harm, and harm to others. This questionnaire was completed with the help of a direct nurse of the elderly.

### 2.5 Statistical analysis

The data were analyzed using the SPSS software (IBM SPSS) version 20. The Kolmogorov−Smirnov test was used for assessing the normality of the data distribution. The chi‐square test and independent sample *t*‐test were used to determine the differences in demographic and clinical variables in men and women (Table 1). Ordinal logistic regression (PLUM model) was used to assess the factors associated with malnutrition (different levels of malnutrition were normally nourished, at risk for malnutrition, and malnourished). Variables with a *p* Value less than 0.02 in Univariate Logistic Regression entered into the Multivariate Logistic Regression. Also, the association between different levels of malnutrition and PPN score as a variable to assess PPNs was assessed by ordinal logistic regression. All tests were 2‐sided and a *p* Value of less than 0.05 was considered statistically significant. The Assel et al.^16^ guidelines were used to interpret and report the results.

**Table 1:** Demographic and clinical characteristics of elderlies living in the nursing homes for the total population as well as gender.

	Total (*n* = 373)	Female (*n* = 221)	Male (*n* = 152)	*p* Value
Marital status *n* (%)	359	216	143	**<0.001** a
Single	141 (39.3)	75 (34.7)	66 (46.2)	
Married	62 (17.3)	28 (13.0)	34 (23.8)	
Widow	129 (35.9)	106 (49.1)	23 (16.1)	
Divorced	27 (7.5)	7 (3.2)	20 (14.0)	
Educational status *n* (%)	343	211	132	**0.04** a
Illiterate	216 (63.0)	146 (69.2)	70 (53.0)	
Elementary school	55 (16.0)	30 (14.2)	25 (18.9)	
High school	27 (7.9)	14 (6.6)	13 (9.8)	
Diploma	37 (10.8)	17 (8.1)	20 (15.2)	
Bachelor	8 (2.3)	4 (1.9)	4 (3.0)	
Insurance *n* (%)	372	220	152	**0.03** a
Yes	265 (70.2)	166 (75.5)	99 (65.1)	
No	107 (29.8)	54 (24.5)	53 (34.9)	
Age (year) (*n* = 342)	75.98 ± 10.74	78.17 ± 10.87	72.61 ± 9.63	**<0.001** b
BMI (kg/m^2^) (*n* = 318)	23.88 ± 4.47	23.61 ± 4.85	24.96 ± 8.43	0.07^b^
Number of children (*n* = 166)	3.48 ± 2.07	3.60 ± 1.90	3.26 ± 2.35	0.31^b^
PPN score (*n* = 338)	28.99 ± 7.84	30.93 ± 7.48	26.51 ± 8.45	**<0.001** b
Calf circumference (cm) (*n* = 256)	21.97 ± 7.23	22.78 ± 8.32	20.63 ± 4.62	**0.008** b
SBP (mmHg) (*n* = 342)	124.12 ± 12.39	123.44 ± 11.83	125.11 ± 13.13	0.23^b^
DBP (mmHg) (*n* = 342)	76.45 ± 7.05	76.07 ± 7.14	77.00 ± 6.90	0.23^b^
FBS (mg/dL) (*n* = 328)	96.56 ± 31.02	98.59 ± 35.60	93.94 ± 23.71	0.17^b^
BUN (mg/dL) (*n* = 296)	16.78 ± 7.12	17.35 ± 7.92	15.97 ± 5.72	**<**0.100^b^
Cr (mg/dL) (*n* = 292)	1.02 ± 0.48	0.98 ± 0.28	1.08 ± 0.66	0.07^b^
TG (mg/dL) (*n* = 303)	128.53 ± 45.49	126.06 ± 49.48	131.68 ± 39.78	0.28^b^
Total‐C (mg/dL) (*n* = 267)	171.90 ± 43.92	174.45 ± 48.62	169.13 ± 38.16	0.32^b^
Hb (g/dL) (*n* = 301)	13.15 ± 1.69	12.80 ± 1.58	13.69 ± 1.73	**<0.001** b

*Note*: Data reported as mean ± SD, otherwise it is stated. *p* Value less than 0.05 considered statistically significant. Significant result are shown in Bold.

Abbreviations: BMI, body mass index; BUN, Blood urea nitrogen; Cr, Creatinine; DBP,: Diastolic blood pressure; FBS, Fasting blood sugar; Hb, Hemoglobin; PPN, Physical and psychological need; SBP, systolic blood pressure; SD, standard deviation; TG, Triglyceride; Total‐c, Total cholesterol.

a Chi‐square test;

^b^ Independent sample *t*‐test.

## 3 RESULTS

A total of 373 elderlies living in nursing homes entered into the study. Two hundred and twenty‐one (59.2%) of them were females. The mean age of the participants was 75.98 ± 10.74 years; which was 72.61 ± 9.63 years in males and 78.17 ± 10.87 years in females (*p* = 0.001). Of the participants, 17% were married at that moment, and 30% were out of any insurance coverage. The mean PPN score was 28.99 ± 7.84, which was significantly different between genders (*p* = 0.001). Table 1 summarizes the demographic and clinical characteristics of participants.

MNA questionnaire was completed for 280 out of 373 elderlies due to their reluctance to participate or severe Alzheimer's. Accordingly, the elderlies were categorized into three groups based on the MNA questionnaire, including normally nourished (*n* = 52, 18.6%), at risk of malnutrition (*n* = 175, 62.5%), and malnourished (*n* = 53, 18.9%). Table 2 shows the results of the univariate ordinal regression reflecting the differences in the demographic indices and clinical statuses of elderlies with regard to their malnutrition conditions. Of the participants, 55% of females and 45% of males were categorized as at risk of malnutrition. The PPN scores of the elderlies at risk of malnutrition and the malnourished were significantly higher than their normally nourished peers (*p* = 0.011). Elderlies with no insurance coverage mainly fell into the at‐risk and malnourished group (*p* = 0.020). Furthermore, 93.5% of elderlies were only using foods prepared by the centers, and malnutrition was not associated with the food supply place of elderlies. The univariate analysis revealed that the participants with varying dental health conditions or adherence to specific diets were not different in their malnutrition conditions. However, there was a significant association between physical strength and malnutrition status (*p* = 0.020). Elderlies lacking regular laboratory documents in their files (86.2%) mainly fell in the at‐risk and malnutrition groups (*p* = 0.03). There were no significant differences among the elderly groups in terms of visiting physicians, nutritionists, and psychologists. Nonetheless, there were significant differences in terms of visiting psychiatrists (*p* = 0.04). Elderlies visiting psychiatrists irregularly or having no psychiatric reports were mainly in the at‐risk and malnutrition groups.

**Table 2:** Factors related to the malnutrition status in elderlies living in the nursing homes: Univariate analysis.

Variables	Total (*n* = 280)	Normally nourished (*n* = 52)	At risk of malnutrition (*n* = 175)	Malnourished (*n* = 53)	*p* Value^a^
Age (year) Mean ± SD	262	79.20 ± 11.11	75.67 ± 10.85	75.02 ± 9.84	**0.05**
BMI (kg/m^2^) Mean ± SD	250	25.99 ± 3.56	23.43 ± 3.87	21.71 ± 5.02	**<0.001**
PPN score Mean ± SD	258	24.84 ± 6.05	28.48 ± 7.70	34.76 ± 6.60	**<0.001**
SBP (mmHg) Mean ± SD	250	123.49 ± 14.60	123.29 ± 10.10	132.64 ± 14.13	**<0.001**
DBP (mmHg) Mean ± SD	250	78.06 ± 7.308	76.66 ± 6.551	80.81 ± 6.784	**0.040**
Gender	280				**0.02**
Male	120 (42.9)	15 (28.8)	78 (44.6)	27 (50.9)	
Female	160 (57.1)	37 (71.2)	97 (55.4)	26 (49.1)	
Marital status	269				0.91
Single (Widowed, Divorced)	227 (84.4)	41 (80.4)	144 (85.7)	42 (84.0)	
Married	42 (15.6)	10 (19.6)	24 (14.3)	8 (16.0)	
Educational status	254				
Illiterate	164 (64.6)	32 (69.6)	98 (62.0)	34 (68.0)	0.38
Elementary school	41 (16.1)	7 (15.2)	27 (17.1)	7 (14.0)	
High school	16 (6.3)	1 (2.2)	12 (7.6)	3 (6.0)	
Diploma	28 (11.0)	4 (8.7)	19 (12.0)	5 (10.0)	
Bachelor	5 (2.0)	2 (4.3)	2 (1.3)	1 (2.0)	
Medical need assessments					
Insurance	279				**0.02**
Yes	212 (76.0)	43 (84.3)	134 (76.6)	35 (66.0)	
No	67 (24.0)	8 (15.7)	41 (23.4)	18 (34.0)	
Providing Food	277				0.96
Nursing home	265 (95.7)	49 (96.1)	165 (95.4)	51 (96.2)	
Home & Nursing home	12 (4.3)	2 (3.9)	8 (4.6)	2 (3.8)	
Appetite	275				**<0.001**
Fair	233 (84.7)	33 (64.7)	151 (88.3)	49 (92.5)	
Moderate	39 (14.2)	15 (29.4)	20 (11.7)	4 (7.5)	
Poor	3 (1.1)	3 (5.9)	0	0	
Special diet	250				0.17
Yes	41 (16.4)	12 (29.3)	21 (51.2)	8 (19.5)	
No	209 (83.6)	33 (15.8)	135 (64.6)	41 (19.6)	
Dental status	248				0.08
Natural teeth	98 (39.5)	14 (30.4)	63 (39.9)	21 (47.7)	
Artificial teeth	89 (35.9)	17 (37.0)	54 (34.2)	18 (40.9)	
No teeth	61 (24.6)	15 (32.6)	41 (25.9)	5 (11.4)	
Walking ability	278				**0.02**
Ability to walk	100 (36.0)	5 (9.6)	71 (41.0)	24 (45.3)	
Partial ability to walk	63 (22.7)	6 (11.5)	41 (23.7)	16 (30.2)	
Not able to walk	52 (18.7)	16 (30.8)	25 (14.5)	11 (20.8)	
Bed bound	63 (22.7)	25 (48.1)	36 (20.8)	2 (3.8)	
Diabetes	226				0.38
Yes	24 (10.6)	3 (6.5)	16 (11.5)	5 (12.2)	
Cardiovascular disease	226				0.11
Yes	65 (28.8)	14 (30.4)	32 (23.0)	19 (46.3)	
Neurologic disorders	225				**0.02**
Yes	137 (60.9)	32 (69.6)	86 (62.3)	19 (46.3)	
Kidney disease	226				**0.02**
Yes	5 (2.2)	0	2 (1.4)	3 (7.3)	
Cancer	226				0.08
Yes	3 (1.3)	2 (4.3)	1 (0.7)	0	
Physical disability	226				**0.05**
Yes	40 (17.7)	11 (23.9)	26 (18.7)	3 (7.3)	
GI disease	284				**0.004**
Yes	8 (3.5)	0	3 (2.2)	5 (12.2)	
Laboratory tests	280				**0.03**
Regular schedule (every 3‐6 m.)	17 (6.1)	5 (9.6)	8 (4.6)	4 (7.5)	
Once a year	123 (43.9)	26 (50)	91 (52.0)	6 (11.3)	
Less than once a year	31 (11.1)	6 (11.5)	20 (11.4)	5 (9.4)	
No lab report in medical documents	109 (38.9)	15 (28.8)	56 (32.0)	38 (71.7)	
Physician visit	280				0.97
Organized schedule	254 (90.7)	46 (88.5)	161 (92.0)	47 (88.7)	
Not‐organized schedule	0	0	0	0	
No report in medical documents	26 (9.3)	6 (11.5)	14 (8.0)	6 (11.3)	
Internist visit	280				0.94
Organized schedule	4 (1.4)	1 (1.9)	3 (1.7)	0	
Not‐organized schedule	9 (3.2)	2 (3.8)	7 (4.0)	0	
No report in medical documents	267 (95.4)	49 (94.2)	165 (94.3)	53 (100)	
Nutritionist visit	280				0.51
Organized schedule	244 (87.1)	46 (88.5)	158 (90.3)	40 (75.5)	
Not‐organized schedule	15 (5.4)	2 (3.8)	9 (5.1)	4 (7.5)	
No report in medical documents	21 (7.5)	4 (7.7)	8 (4.6)	9 (17.0)	
Psychologist visit	280				0.15
Organized schedule	271 (96.8)	51 (98.1)	171 (97.7)	49 (92.5)	
Not‐organized schedule	4 (1.4)	1 (1.9)	2 (1.1)	1 (1.9)	
No report in medical documents	5 (1.8)	0	2 (1.1)	3 (5.7)	
Psychiatrists visit	280				**0.04**
Organized schedule	122 (43.5)	31 (59.6)	84 (48.1)	7 (13.2)	
Not‐organized schedule	7 (2.5)	2 (3.8)	5 (2.9)	0	
No report in medical documents	151 (53.9)	19 (36.5)	86 (49.1)	46 (86.8)	

*Note*: Data reported as *N* (%), otherwise it is stated. *p* Value less than 0.05 considered statistically significant. Significant result are shown in Bold.

Abbreviations: BMI, body mass index; DBP, Diastolic blood pressure; GI, gastrointestinal; PPN, Physical and psychological need; SBP, systolic blood pressure; SD, standard deviation.

^a^ Univariate Ordinal Regression.

The association between elderlies' malnutrition status and their PPN score was investigated by multivariate ordinal regression analysis. Several variables, including BMI, Walking ability, appetite, and calf circumference, which are the MNA components for investigating the malnutrition status, were excluded from the analysis. There was a significant relationship between the needs assessment scores, reflecting the need for physical and psychological actions, and elderlies' malnutrition (odds ratio [OR]: 1.87, 95% confidence interval [CI]: 1.79−1.95). For each unit increase in the PPN score, the odds of exposure to malnutrition (vs. normally nourished and at risk of malnutrition) was 1.87 times after adjusting for confounders. According to the results, for a one mmHg increment in the systolic blood pressure, the odds of suffering from malnutrition became 1.09 times (OR: 1.09, 95% CI: 1.03−1.16). For participants suffering from neurological diseases, the odds of malnutrition (vs. normally nourished and at‐risk ones) were 3.18 times higher than for elderlies with no neurological disorders. Adherence to a special dietary regimen raised the odds of malnutrition in comparison with normally nourished and at‐risk individuals by 2.70 times. Table 3 displays the results of the multivariate analysis.

**Table 3:** Factors related to the malnutrition status in elderlies living in the nursing homes: Multivariate analysis.

	OR	CI 95% for OR	*p* Value^a^
PPN score	1.87	1.79−1.95	**0.003**
Age	1.02	0.97−1.07	0.38
SBP	1.09	1.03−1.16	**0.003**
DBP	0.94	0.86−1.03	0.83
Gender			
Male	Ref	‐	‐
Female	1.23	0.33−4.58	0.74
Insurance			
No	Ref	‐	‐
Yes	0.58	0.43−4.23	0.27
Cardiovascular disease			
No	Ref	‐	‐
yes	1.09	0.27−4.38	0.34
Neurologic drug use			
No	Ref	‐	‐
Yes	3.18	0.96−10.50	**0.05**
Kidney disorders			
No	Ref	‐	‐
Yes	1.97	0.02−132.04	0.81
Cancer			
No	Ref	‐	‐
Yes	4.98	0.07−32.54	0.40
Physical disability			
No	Ref	‐	‐
Yes	2.39	0.44−12.88	0.30
GI problem			
No	Ref	‐	‐
Yes	1.37	0.01−11.13	0.11
Regular lab test			
Regular schedule (every 3−6 m.)	Ref	‐	‐
Once a year	0.15	0.001−2.67	0.29
Less than once a year	0.87	0.11−6.64	0.89
No lab report in medical documents	1.84	0.26−9.33	0.44
Psychologist visit			
Regular visits	Ref	‐	‐
Irregular visits	1.03	0.003−14.20	0.70
No report	1.01	0.04−18.80	0.90
Psychiatrist visit			
Regular visits	Ref	‐	‐
Irregular visits	1.18	0.18−12.25	0.60
No reports	2.08	0.29−14.64	0.46
Being on special diet			
No	Ref	‐	‐
Yes	2.70	2.37−8.65	**0.006**

*Note*: *p* Value less than 0.05 considered statistically significant. Significant result are shown in Bold.

Abbreviations: CI, confidence interval; DBP, diastolic blood pressure; GI, gastrointestinal; PPN, physical and psychological need; SBP, systolic blood pressure; OR, odds ratio.

a Multivariate ordinal regression.

**Figure 1:**
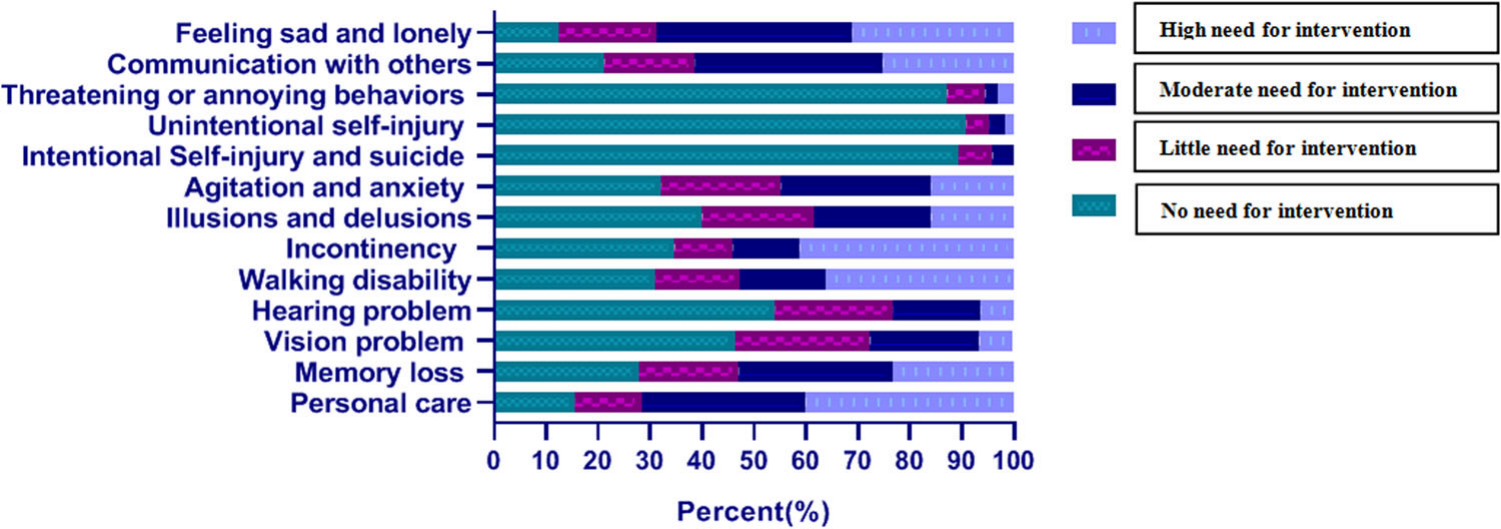
The need for intervention to solve physical and psychological problems.

Figure 1 illustrates the caring nurses' perspectives on the need for intervention for the solution of elderlies' physical and psychological problems given different variables, including visual, hearing, communicational, memory, and so forth, impairments.

## 4 DISCUSSION

The present cross‐sectional study investigated elderlies' nutritional and health status and their possible relationship with the care services they received in Shiraz, Iran's nursing homes. The results of this study showed that the physical and psychiatric needs of the elderly who are at risk of malnutrition and malnourished ones are significantly higher.

The PPN phenomenon investigates two major aspects potentially related to malnutrition: first, psychiatric problems, such as depression, loneliness, and other mental‐related disorders which can cause appetite loss and ultimately lead to malnutrition among elderlies. In line with our study, it was seen depression,^17^ and feelings of loneliness^18^ as the components of psychiatric disorders that increase malnutrition risk. Experiencing any kind of abuse among elderlies affects their health.^19^ In a study in Iran, it was shown that nursing home dwellers elderlies with lower mental health and social relations scores have a worsened nutritional status than other elderly.^20^

Second, physical needs may enhance the risk of malnutrition. The study done by Ning et al.^21^ supports our results, in that elderlies with higher physical function dependency are more prone to be malnourished. However, a study in Tabriz, Iran, did not see any correlation between physical health and nutritional status in the elderly living in nursing houses.^20^

Healthcare costs for managing malnutrition are high.^22^ Our finding indicates the percentage of malnourished elderlies with insurance coverage was lower than the percentage of normally nourished ones. Our result supported a finding by Wei et al.^23^ which showed a higher prevalence of malnutrition among elderlies with a lack of insurance coverage. This can be explained by that without health insurance coverage, the use of healthcare services is lower.^24^ In the study done by Khajeh et al.^24^ it was seen that in the absence of health insurance, specialist visits are among the lowest healthcare services that individuals seek.

Other health‐related and demographic statuses of the elderly may be associated with malnutrition. The results of our study disclosed that the ability for physical activity was significantly related to their malnutrition status, which was in line with previous study.^12^ Liguori et al.^25^ showed the MNA score is related to muscle mass (*r* = 0.72), and muscle strength (*r* = 0.42). Thus, more physically active elderlies are less prone to be malnourished. It also can be concluded based on the results seen by Dehdari et al.^26^ that the non‐needing physical rehabilitation services elderlies had better nutritional conditions than their peers in need of physical rehabilitation. Hence, it is denoted that physical activity can positively contribute to supplying the nutritional health of the elderly.

The psychiatrist is among the specialists who are needed by elderlies. The results of the present investigation revealed the association of malnutrition with psychological problems. Psychiatric and anorexic impairments may complicate the marginal conditions for the malnutrition risk among old people, who are susceptible to psychiatric problems. Noticeably, this subject is a paramount problem for many elderlies as well.^27^, ^28^ It can be asserted that suffering from psychiatric problems is a risk factor for malnutrition, which is not diagnosed and controlled in 70% of acute hospitalizations. Hence, we can consider psychiatric problems as predictors of malnutrition.^29^ Therefore, the regular visits of elderlies by psychologists and psychiatrists, besides the early diagnosis and treatment of psychological disorders, not only improve patients' mental statuses but also decrease the malnutrition risk and its associated problems.

According to the results of our study, elderlies having a physical illness or who follow a special medical diet had experienced lower nutritional status. Elderlies have a variety of physiological needs. Malnutrition is a prevalent undesirable health outcome in the elderly. It seems regular nutritionist and dietitian visits may prevent malnutrition. Thus enhancing diet can benefit elderlies and help them to meet their needs. A previous systematic review revealed that malnutrition prevention and management might have a better outcome when other health providers have enough nutritional knowledge and understanding of the importance of nutritional interventions in malnutrition.^30^ Thus, health providers should be informed about the importance of nutritionists visiting the elderly regularly.

A practical and realistic approach to optimize food intake could help elderlies facing malnutrition. There is a controversy about protein requirements in the elderly.^31^ However, increasing the variety of diets, their palatability, and amendments in flavor may help increments in intake and effects of medical nutrition therapy.^31^ Thus planning a cost‐effective diet, based on the elderly's interests and preferences may be the cornerstone in managing malnutrition. In addition, a study conducted in Canada demonstrated that a higher number and types of medication elderlies take might reduce the rate of their nourishment,^32^ which should be taken into account.

## 5 LIMITATION

Although the present study was among the few surveys that inclusively addressed the health of the elderly by focusing on their nutritional status, it faced limitations including the high prevalence of memory impairments and Alzheimer's disease (>70%) among the elderly in nursing homes and conducting the study during the Covid‐19 outbreak. These issues impeded the completion of the questionnaires by the elderlies themselves but with the assistance of their nurses. The next limitation was that the information might have been incompletely recorded in the files of some centers. Therefore, the elderlies might have received some unrecorded services, an issue that might indicate the irregularity of service provision.

## 6 CONCLUSION

The results of our study revealed that some levels of malnutrition were prevalent in the nursing homes of Shiraz, such as being at risk of malnutrition (62.5%) or being malnourished (18.9%). Individuals who suffer from different levels of malnutrition encompassed approximately half of the residing population with outnumbering physical and psychological health needs. Also, this study showed that the elderly being exposed to regular physical and psychiatric care generally possessed better nutritional conditions. Based on these results, it is, first, suggested that policymakers in the health and well‐being domain take practical steps toward promoting care fitted with the (nutritional) needs of old people and direct extra supervision to nursing homes and care provisions. Second, it is recommended that more accurate studies be conducted in combination with evaluating the physical, mental, and nutritional health of the elderly after control of the Covid‐19 pandemic.

## AUTHOR CONTRIBUTIONS

PK was resposible for the study concept and design, data interpretation, drafting the manuscript, critical revision of the manuscript and study supervision. HJ and MF were responsible for the study concept and design, data interpretation, critical revision, administrative support, and study supervision. MAM and SMK were responsible for data collection, data interpretation, drafting the manuscript and critical revision. ZF was responsible for data analysis, data interpretation, drafting of the manuscript, and critical revision of the manuscript.

## CONFLICT OF INTEREST STATEMENT

The authors declare no conflict of interest.

## ETHICS STATEMENT

The Ethical Committee of Deputy for Research affiliated with Shiraz University of Medical Sciences provided ethical clearance (IR.SUMS.REC.1399.1046) for this study. For the data that had to be collected directly from participants, they were informed about the study objectives and then asked to sign the informed consent before the interview. The interviews were conducted by respectful nurses and in a private place.

## TRANSPARENCY STATEMENT

The lead author Parisa Keshani affirms that this manuscript is an honest, accurate, and transparent account of the study being reported; that no important aspects of the study have been omitted; and that any discrepancies from the study as planned (and, if relevant, registered) have been explained.

## Data Availability

The data used to support the findings of this study are available from the corresponding author upon request.
